# Leukemia inhibitory factor regulates Schwann cell proliferation and migration and affects peripheral nerve regeneration

**DOI:** 10.1038/s41419-021-03706-8

**Published:** 2021-04-22

**Authors:** Qianqian Chen, Qianyan Liu, Yunsong Zhang, Shiying Li, Sheng Yi

**Affiliations:** 1 NMPA Key Laboratory for Research and Evaluation of Tissue Engineering Technology Products, Key Laboratory of Neuroregeneration of Jiangsu and Ministry of Education, Co-innovation Center of Neuroregeneration, Nantong University, Nantong, Jiangsu 226001 China; 2 State Key Laboratory of Pharmaceutical Biotechnology and MOE Key Laboratory of Model Animal for Disease Study, Model Animal Research Center, Nanjing Biomedical Research Institute, Nanjing University, Nanjing, China

**Keywords:** Cell migration, Regeneration and repair in the nervous system

## Abstract

Leukemia inhibitory factor (LIF) is a pleiotropic cytokine that stimulates neuronal development and survival. Our previous study has demonstrated that LIF mRNA is dysregulated in the peripheral nerve segments after nerve injury. Here, we show that LIF protein is abundantly expressed in Schwann cells after rat sciatic nerve injury. Functionally, suppressed or elevated LIF increases or decreases the proliferation rate and migration ability of Schwann cells, respectively. Morphological observations demonstrate that in vivo application of siRNA against LIF after peripheral nerve injury promotes Schwann cell migration and proliferation, axon elongation, and myelin formation. Electrophysiological and behavior assessments disclose that knockdown of LIF benefits the function recovery of injured peripheral nerves. Differentially expressed LIF affects the metabolism of Schwann cells and negatively regulates ERFE (Erythroferrone). Collectively, our observations reveal the essential roles for LIF in regulating the proliferation and migration of Schwann cells and the regeneration of injured peripheral nerves, discover ERFE as a downstream effector of LIF, and extend our understanding of the molecular mechanisms underlying peripheral nerve regeneration.

## Introduction

Peripheral nerve injury impairs the communication between central nerves with muscles and organs, affects about 2.8% trauma patients, and induces weakness, tingling, numbness, motorial and/or sensory deficits^1,2^. Although injured peripheral nerves are able to heal themselves, the functional recovery of severe or chronic peripheral nerve injury is generally disappointing. Since the discovery of nerve growth factor (NGF) as a robust stimulator of neuron survival and axonal sprouting in 1951^3,4^, neurotrophic factors have been identified as critical modulating factors for peripheral nerve regeneration^5,6^.

Schwann cells are unique glial cells in the peripheral nervous system and main population cells in peripheral nerves^7^. Following peripheral nerve injury, Schwann cells undergo phenotypic changes, proliferate to restore lost cell populations, and migrate from injured nerve segments to build a regeneration path^8,9^. Therefore, satisfying functional restoration of injured peripheral nerves requires not only the activation of the intrinsic growth capacity of neurons, but the joint work of Schwann cells^10^. Emerging studies have shown that besides neurons, neurotrophic factors can also influence Schwann cell behaviors. For instance, Schwann cells transfected with siRNA against NGF or brain derived neurotrophic factor (BDNF) exhibit suppressed proliferation and migration rates, indicating the promoting roles of NGF and BDNF on Schwann cell proliferation and migration^11,12^.

Previously, we have screened differentially expressed genes in rat sciatic nerve segments after peripheral nerve injury, discovered enriched Gene Ontology (GO) terms, and found that LIF, a gene encoding leukemia inhibitory factor (LIF), is involved in the GO term “negative regulation of cell proliferation”^13^. LIF is a neurotrophic factor that supports the development and survival of neurons and rescues neuronal death after axotomy^14–17^. Despite the protecting roles of LIF on neurons, the biological functions of LIF on Schwann cells remains largely unrevealed.

In the current study, we cultured Schwann cells, transfected Schwann cells with siRNA against LIF or lentivirus overexpressing LIF, and examined the effect of LIF on Schwann cell phenotype. The biological roles of LIF on peripheral nerve regeneration and downstream factor(s) of LIF were also examined.

## Results

### LIF modulates Schwann cell proliferation and migration

Our previous study has examined the mRNA abundance of LIF in the injured rat sciatic nerve segments and demonstrated that LIF mRNA expression is upregulated at 1 day, 4 days, and 7 days after peripheral nerve injury and gradually recovered to the normal level at 14 days after nerve injury^13^. Rat sciatic nerve segments were further subjected to immunostaining to determine the protein abundance of LIF after nerve injury. Immunostaining observations showed that, consistent with gene expression patterns, the expression levels of LIF protein seemed to be elevated after nerve injury (Fig. S1). Moreover, LIF protein was observed to be co-localized with Schwann cell marker S100β, indicating that LIF is expressed in Schwann cells and may affect Schwann cell phenotype.

**Fig. 1:**
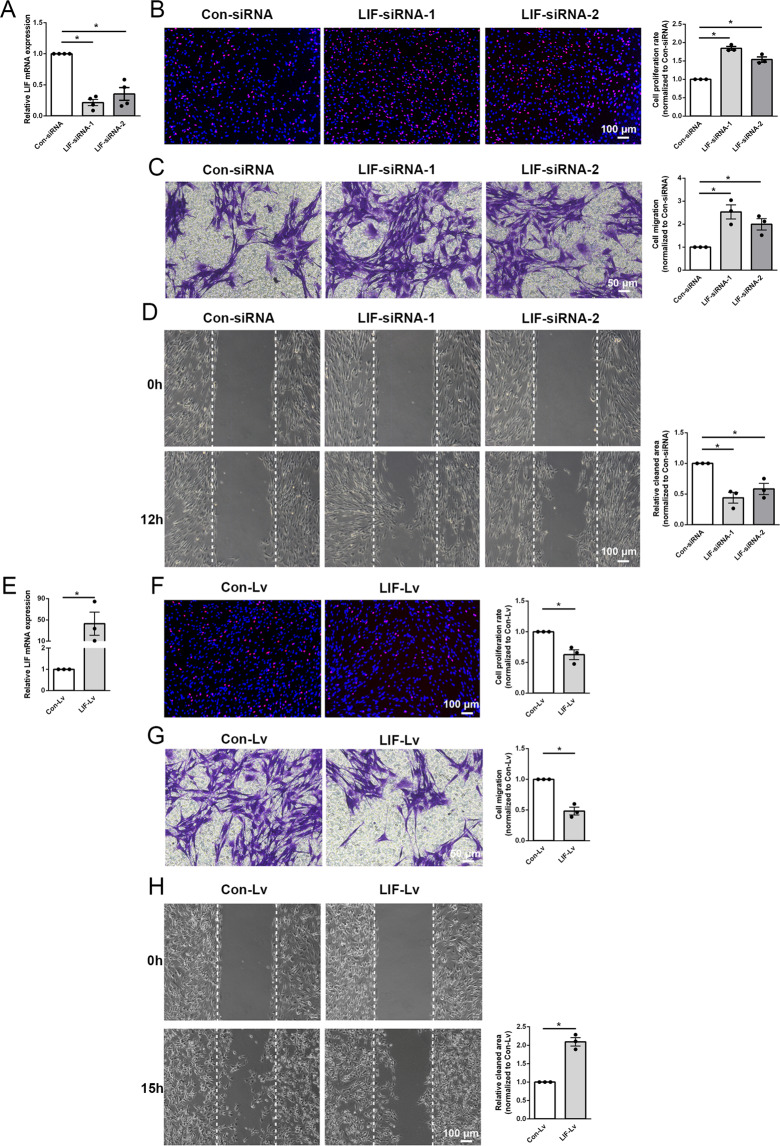
LIF affects Schwann cell proliferation and migration. **A** Transfection of Schwann cells with LIF-siRNA-1 or LIF-siRNA-2 reduces the mRNA abundance of LIF. **B** LIF-siRNA-1 or LIF-siRNA-2 transfection increases Schwann cell proliferation. Red color indicates EdU-positive cells and blue color indicates nucleus. Scale bar indicates 100 μm. **C** LIF-siRNA-1 or LIF-siRNA-2 transfection increases Schwann cell migration. Violet color indicates migrated cells. Scale bar indicates 50 μm. **D** LIF-siRNA-1 or LIF-siRNA-2 transfection decreases cleaned areas between scratches. Scale bar indicates 100 μm. **E** Transfection of Schwann cells with LIF-overexpressing lentivirus (LIF-Lv) reduces the mRNA abundance of LIF. **F** LIF-overexpressing lentivirus transfection decreases Schwann cell proliferation. Red color indicates EdU-positive cells and blue color indicated nucleus. Scale bar indicates 100 μm. **G** LIF-overexpressing lentivirus transfection decreases Schwann cell migration. Violet color indicates migrated cells. Scale bar indicates 50 μm. **H** LIF-overexpressing lentivirus transfection increases cleaned areas between scratches. Scale bar indicates 100 μm. Data were presented as means ± SEM. *p value <0.05 versus control-siRNA (Con-siRNA) or control-lentivirus (Con-Lv).

To evaluate the effects of LIF on Schwann cells, Schwann cells were transfected with siRNA segments against LIF to knockdown LIF expression. Transfection with both LIF-siRNA-1 and LIF-siRNA-2 robustly reduced the mRNA abundance of LIF (Fig. 1A). EdU assay demonstrated that inhibition of LIF expression increased the number of EdU-positive Schwann cells and led to elevated proliferation rate (Fig. 1B). Transwell migration assay showed that LIF knockdown promoted the migration of Schwann cells through the Transwell chamber (Fig. 1C). Similarly, wound healing assay showed that compared with Schwann cells transfected with siRNA control, Schwann cells transfected with LIF-siRNA-1 and LIF-siRNA-2 obtained greater migration ability towards the scratch wound, leaving a relatively smaller cleaned area (Fig. 1D).

Schwann cells were further transfected with LIF-overexpressing lentivirus to determine the effect of overexpressed LIF on cell proliferation and migration (Fig. 1E). Contrary to cells transfected with LIF-siRNA, Schwann cells transfected with LIF-overexpressing lentivirus exhibited reduced proliferation rate (Fig. 1F) and suppressed migration ability (Fig. 1G, H). These findings suggest that LIF inhibits the proliferation and migration of Schwann cells.

### LIF regulates cellular behaviors after peripheral nerve injury

**Fig. 2:**
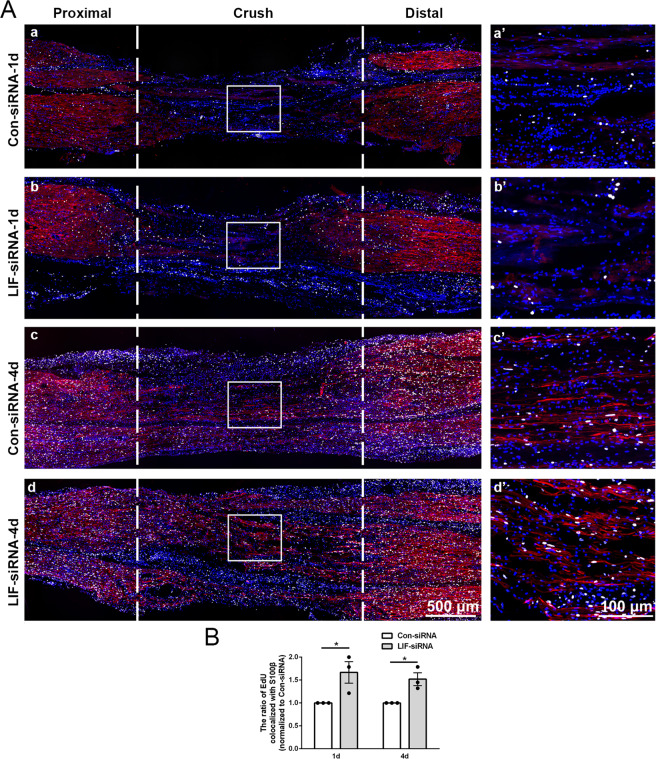
LIF-siRNA promotes Schwann cell proliferation in vivo. **A** Representative immunostaining images of sciatic nerve longitude sections of rats injected with control-siRNA or LIF-siRNA. Rat sciatic nerve segments are collected at (a and b) 1 day and (c and d) 4 days after nerve crush injury. Injured areas (proximal segments, crush sites, and distal segments) are labeled with dashed lines. White color indicates Ki67 staining of proliferating cells, red color indicates S100β, and blue color indicates nucleus. Boxed areas are demonstrated at a higher magnification on the right. Scale bars indicate 500 μm in main images and 100 μm in magnified images. **B** Quantification of the relative number of proliferating Schwann cells in rats injected with control-siRNA or LIF-siRNA at 1 day and 4 days after nerve crush injury. Data were presented as means ± SEM. *p value <0.05 versus control-siRNA (Con-siRNA).

The in vivo effect of LIF on Schwann cell proliferation and migration after peripheral nerve injury was evaluated by injecting LIF-siRNA to the injured sites of rat peripheral nerves. Certain amount of proliferating cells was observed at the crush site at 1 day after nerve injury and a larger number of proliferating cells was detected at a later time point. Many of these proliferating cells were co-labeled with S100β, indicating that Schwann cells are proliferating at the injured nerve segments (Fig. 2A). Compared with rats injected with siRNA control, the number of proliferating Schwann cells was obviously larger in rats injected with LIF-siRNA, suggesting that LIF-siRNA promotes the proliferation of Schwann cells after peripheral nerve injury (Fig. 2B).

**Fig. 3:**
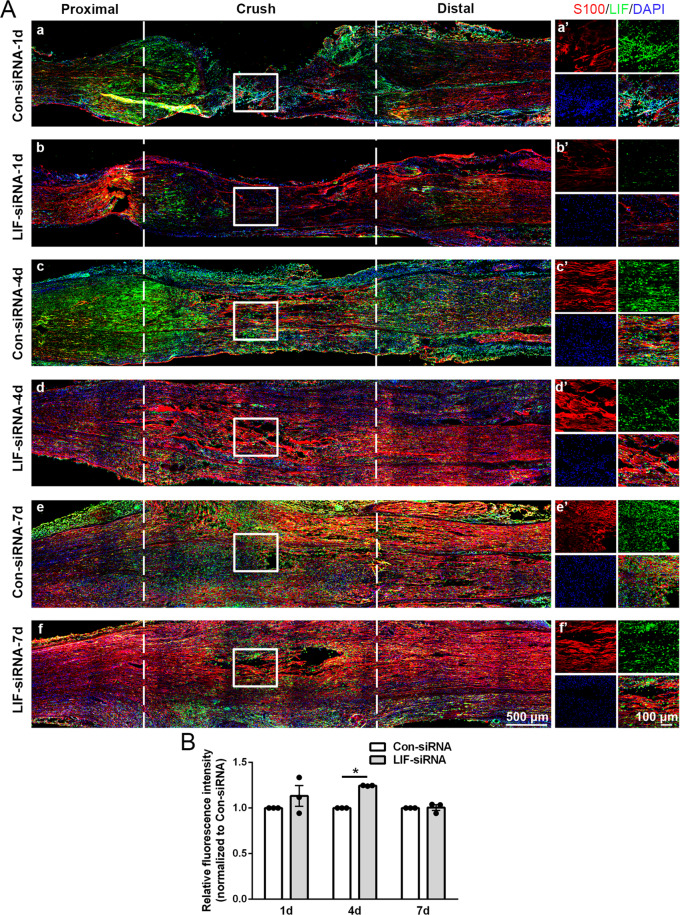
LIF-siRNA promotes Schwann cell migration in vivo. **A** Representative immunostaining images of sciatic nerve longitude sections of rats injected with control-siRNA or LIF-siRNA. Rat sciatic nerve segments are collected at (a and b) 1 day, (c and d) 4 days, and (e and f) 7 days after nerve crush injury. Injured areas (proximal segments, crush sites, and distal segments) are labeled with dashed lines. Green color indicates LIF, red color indicates S100β, and blue color indicates nucleus. Boxed areas are demonstrated at a higher magnification on the right. Scale bars indicate 500 μm in main images and 100 μm in magnified images. **B** Quantification of the relative fluorescence intensity of S100β at the middle of the injured site in rats injected with control-siRNA or LIF-siRNA at 1 day, 4 days, and 7 days after nerve crush injury. Data were presented as means ± SEM. *p value <0.05 versus control-siRNA (Con-siRNA).

Immunostaining of S100β in sciatic nerve segments showed that Schwann cells migrated from both proximal and distal segments to the crush sites after nerve injury (Fig. 3A). A much lower intensity of LIF and higher intensity of S100β as well as a longer migrating distance of Schwann cells were observed in rats injected with LIF-siRNA (Fig. 3A), indicating that reduced LIF expression encourages Schwann cell migration. Schwann cells migrated from the proximal and distal segments were converged and connected in both groups at later time points. But the fluorescence intensity of S100β at the middle of the crushed site was still much higher in rats injected with LIF-siRNA at 4 days after nerve injury (Fig. 3B). In addition, the arrangement of migrated Schwann cells was more neat and ordered at 4 days and 7 days after nerve injury. These observations indicate that the application of LIF-siRNA stimulates the migration of Schwann cells towards the injured site and benefits the formation of a nerve bridge.

**Fig. 4:**
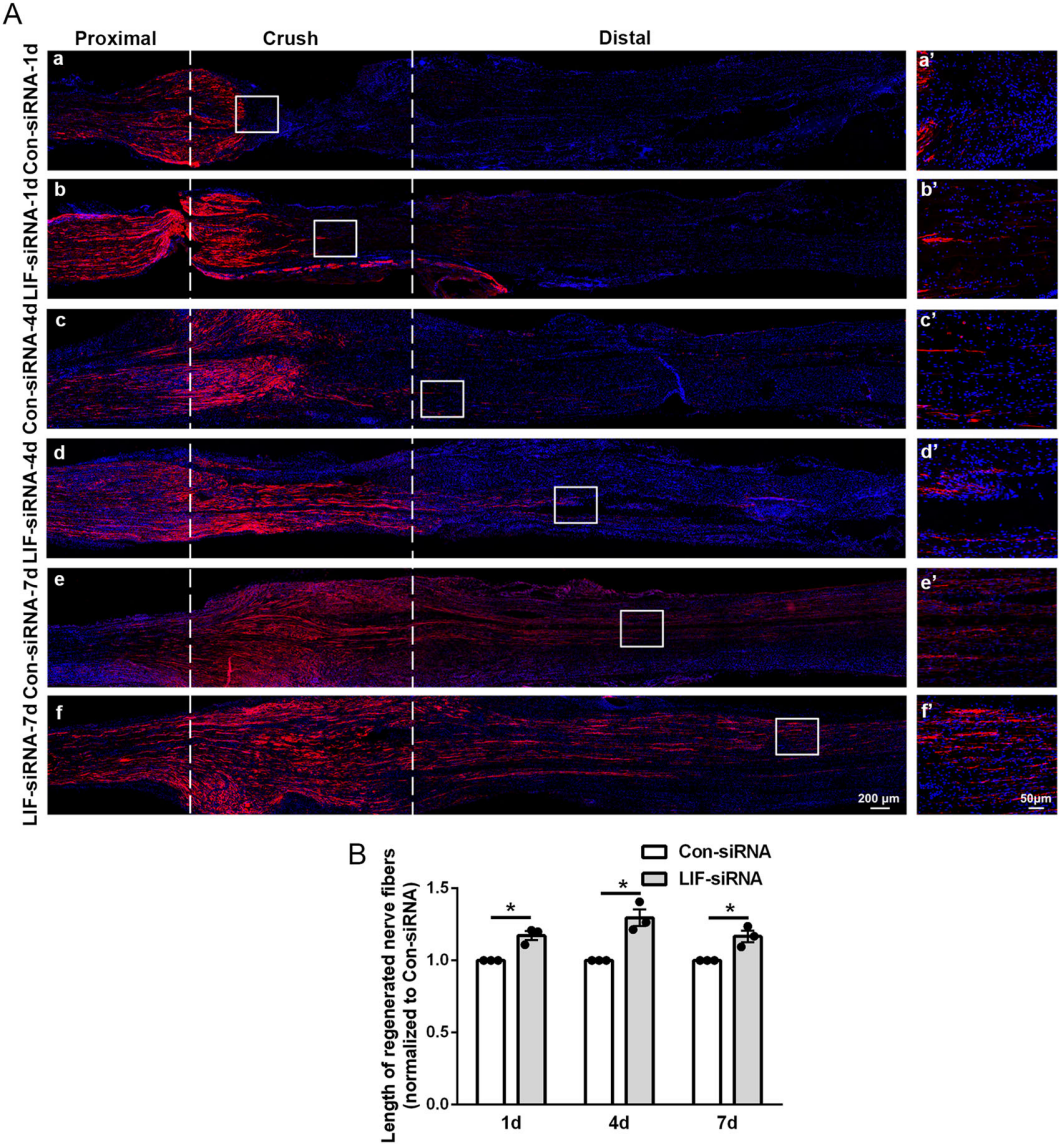
LIF-siRNA promotes axon elongation. **A** Representative immunostaining images of sciatic nerve longitude sections of rats injected with control-siRNA or LIF-siRNA. Rat sciatic nerve segments are collected at (a and b) 1 day, (c and d) 4 days, and (e and f) 7 days after nerve crush. Injured areas (proximal segments, crush sites, and distal segments) are labeled with dashed lines. Red color indicates SCG10 and blue color indicates nucleus. Boxed areas are demonstrated at a higher magnification on the right. Scale bars indicate 200 μm in main images and 50 μm in magnified images. **B** Quantification of the relative length of elongated axons in rats injected with control-siRNA or LIF-siRNA at 1 day, 4 days, and 7 days after nerve crush injury. Data were presented as means ± SEM. *p value <0.05 versus control-siRNA (Con-siRNA).

Subsequently, the growth and elongation of injured axons were evaluated by the immunostaining of regenerating axon marker SCG10. In rats injected with siRNA control, injured axons grow at a normal speed and elongated for about 1 mm at 1 day after nerve crush (Fig. 4A). Compared with rats injected with siRNA control, rats injected with LIF-siRNA had longer axons at 1 day, 4 days, and 7 days after nerve injury (Fig. 4B). These observations, collectively, suggest that knockdown of LIF stimulates the proliferation and migration of Schwann cells after nerve injury, guides axon growth along nerve bridge, and thus enhances peripheral nerve regeneration.

**Fig. 5:**
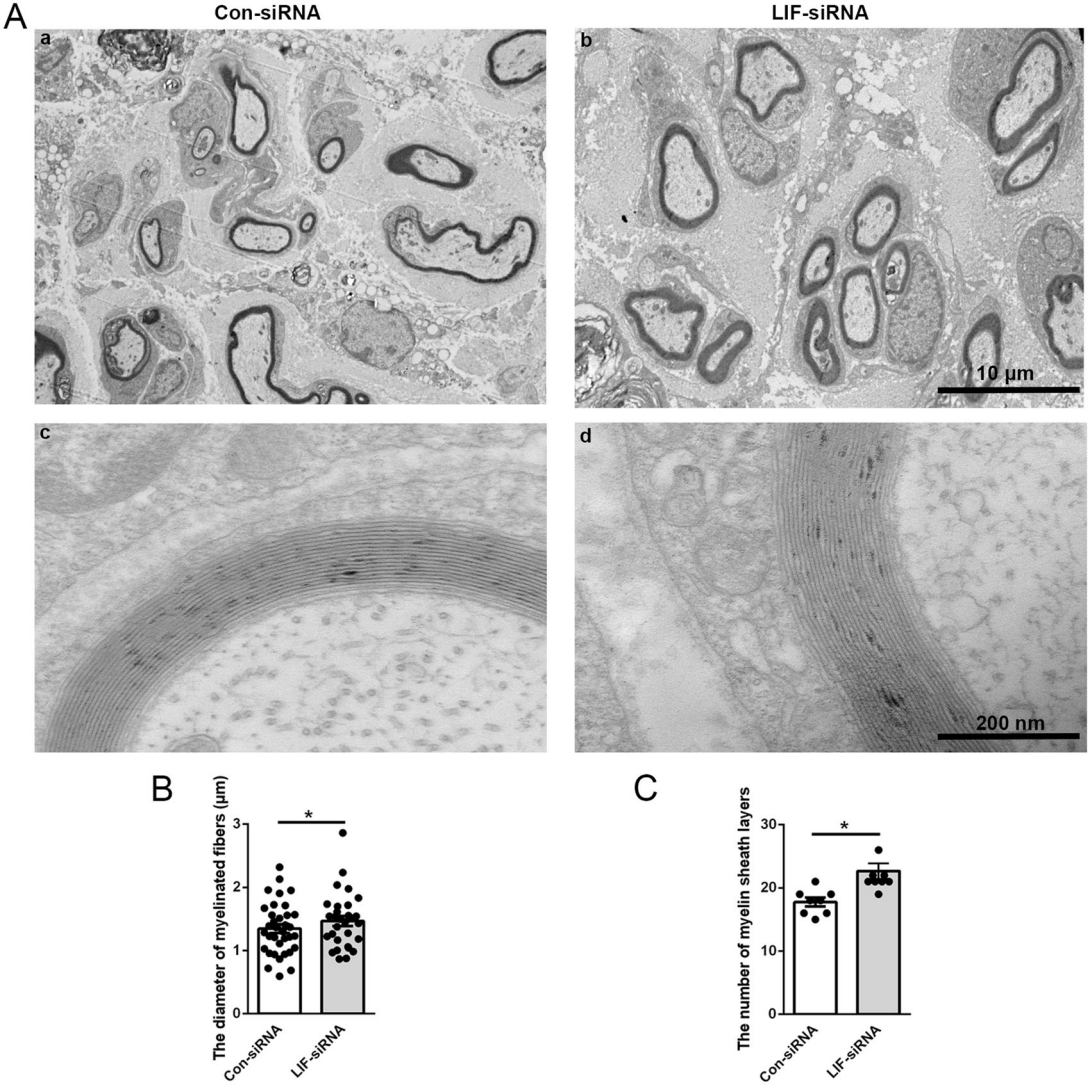
LIF-siRNA promotes myelin sheath formation. **A** Representative transmission electron microscopy images of myelinated nerve fibers of rats injected with control-siRNA or LIF-siRNA at 2 weeks after nerve crush injury. Scale bars indicate 10 μm in upper images (a and b) and 200 nm in lower images (c and d). **B**, **C** Quantification of **B** the diameter of myelinated nerve fibers and **C** the number of myelin sheath layers in rats injected with control-siRNA or LIF-siRNA at 2 weeks after nerve crush injury. Data were presented as means ± SEM. *p value <0.05 versus control-siRNA (Con-siRNA).

Transmission electron micrography images revealed that Schwann cells form myelin sheaths around regenerated axons at 2 weeks after nerve injury (Fig. 5A). Much thicker and compacter myelin sheaths were observed in rats injected with LIF-siRNA. The diameter of myelinated fibers and the number of myelin sheath layers were relatively larger in rats injected with LIF-siRNA as well (Fig. 5B, C). These observations indicate that knockdown of LIF promotes the formation of myelin sheaths and the wrapping of regenerated axons.

### LIF influences the functional recovery of injury peripheral nerves

**Fig. 6:**
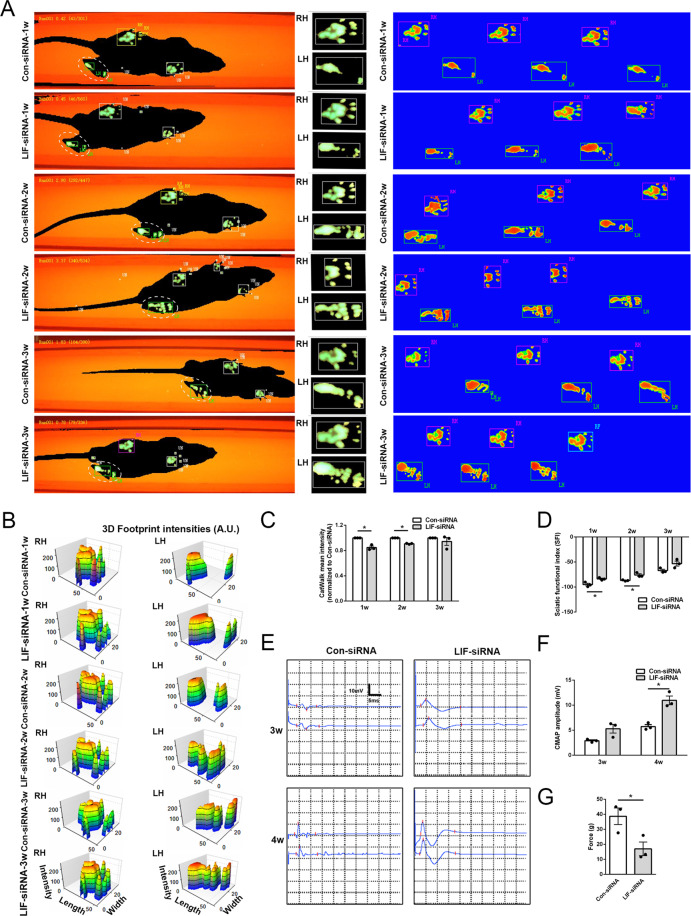
LIF-siRNA stimulates nerve functional recovery. **A** Representative footprint images of rats injected with control-siRNA or LIF-siRNA at 1 week, 2 weeks, and 3 weeks after nerve crush injury. LH indicates the left hindpaw (injured site) and RH indicates right hindpaw (uninjured site). **B** Measurement of 3D footprint intensities of rats injected with control-siRNA or LIF-siRNA at 1 week, 2 weeks, and 3 weeks after nerve crush injury. **C** Quantification of relative CatWalk mean intensity of rats injected with control-siRNA or LIF-siRNA at 1 week, 2 weeks, and 3 weeks after nerve crush injury. **D** Quantification of SFI of rats injected with Control-siRNA or LIF-siRNA at 1 week, 2 weeks, and 3 weeks after nerve crush injury. **E** Representative CMAP recordings of rats injected with control-siRNA or LIF-siRNA at 3 weeks and 4 weeks after nerve crush injury. **F** Quantification of peak amplitude in rats injected with control-siRNA or LIF-siRNA at 3 weeks and 4 weeks after nerve crush injury. **G** Quantification of response to mechanical allodynia in rats injected with control-siRNA or LIF-siRNA at 4 weeks after nerve crush injury. Data were presented as means ± SEM. *p value <0.05 versus control-siRNA (Con-siRNA).

Rat injected with LIF-siRNA were further subjected to CatWalk analysis to examine whether modulation of LIF expression would affect limb coordination. Recorded gait patterns showed that the motor function of nerve-injured rats was gradually recovered (Fig. 6A). The measurement of the intensities of rat left hindpaw (injured site) and right hindpaw (uninjured site) demonstrated that in LIF-siRNA-injected rats, the force and touch of the left hindpaw were more close to that of the right hindpaw (Fig. 6B). CatWalk mean intensity was relatively smaller in rats injected with LIF-siRNA (Fig. 6C), suggesting that in LIF-siRNA injected rats, the hindpaw at the injured site performs similar behaviors as the hindpaw on the uninjured site. Sciatic function index (SFI) values of LIF-siRNA injected rats were also much higher than the SFI value of siRNA-control injected rats (Fig. 6D), indicating that knockdown of LIF alleviates gait alterations.

Compound muscle action potential (CMAP) recordings showed that peak amplitudes could be detected after the application of electrical stimuli. The amplitudes were much higher in LIF-siRNA injected rats as compared with siRNA-control injected rats (Fig. 6E, F), indicating that knockdown of LIF benefits the recovery of nerve conductance. The measurement of mechanical pain thresholds showed that knockdown of LIF modulated the responses to mechanical allodynia (Fig. 6G). These functional assessments suggest that the application of LIF-siRNA after nerve injury improves the recovery of electrophysiological parameters, motor functions, and sensory functions.

### LIF affects Schwann cell metabolism and negatively regulated ERFE

**Fig. 7:**
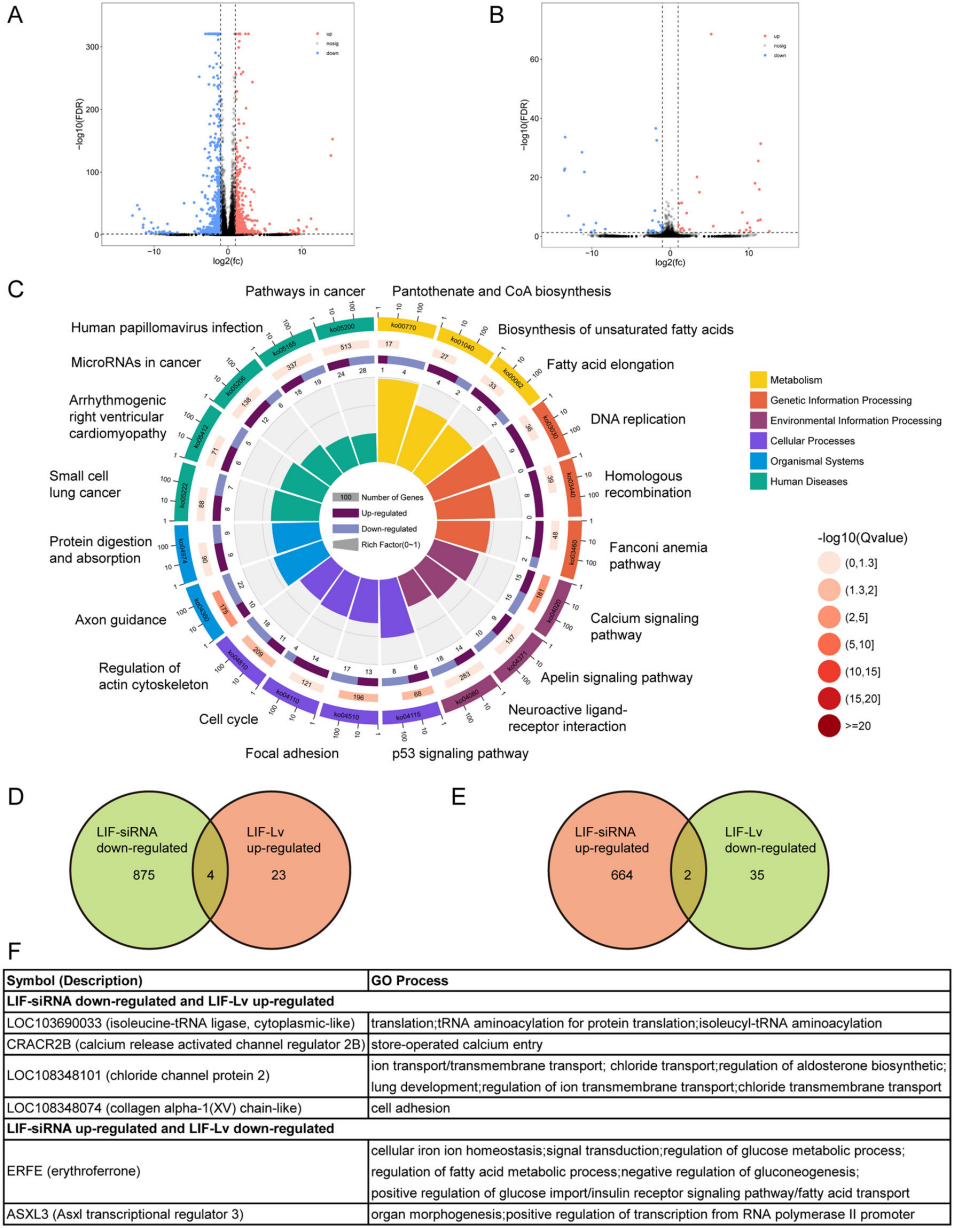
LIF mediates gene expression changes in Schwann cells. **A**, **B** Scatter plots of differentially expressed genes in Schwann cells transfected with (**A**) LIF-siRNA or (**B**) LIF-overexpressing lentivirus (LIF-Lv). Red color indicates significantly upregulated genes and blue color indicates significantly downregulated genes. **C** Top 20 Enriched KEGG pathways of differentially expressed genes in LIF-siRNA-transfected Schwann cells. The length of bar graph in the innermost circle is correlated with the Rich factor of KEGG pathway. **D** Venn diagram of downregulated genes in LIF-siRNA-transfected Schwann cells and upregulated genes in LIF-overexpressing lentivirus-transfected Schwann cells. **E** Venn diagram of upregulated genes in LIF-siRNA-transfected Schwann cells and downregulated genes in LIF-overexpressing lentivirus-transfected Schwann cells. **F** The symbol, description, and GO process of overlaid differentially expressed genes.

To decode LIF-induced molecular mechanism, Schwann cells transfected with LIF-siRNA or LIF-overexpressing lentivirus were subjected to RNA sequencing. A total of 666 and 881 genes were found to be upregulated and downregulated, respectively, in LIF-siRNA-transfected Schwann cells (Fig. 7A). The number of significantly different expressed genes were less in LIF-overexpressing lentivirus-transfected Schwann cells, with 27 upregulated genes and 37 downregulated genes (Fig. 7B). Biological analysis enriched the large number of different expressed genes in LIF-siRNA-transfected Schwann cells to many metabolic, genetic, environmental, cellular, and developmental processes. Pantothenate and CoA biosynthesis, a metabolism-associated Kyoto Enrichment of Genes and Genomes (KEGG) pathway, displayed the highest rich factor (Fig. 7C).

Differentially expressed genes in LIF-siRNA-transfected Schwann cells and LIF-overexpressing lentivirus-transfected Schwann cells were jointly analyzed (Fig. 7D, E). A total of four genes, including LOC103690033, CRACR2B, LOC108348101, and LOC108348074, were found to be downregulated in LIF-siRNA-transfected Schwann cells and upregulated in LIF-overexpressing lentivirus-transfected Schwann cells. A total of two genes, i.e., ERFE and ASXL3, were found to be upregulated in LIF-siRNA-transfected Schwann cells and downregulated in LIF-overexpressing lentivirus-transfected Schwann cells (Fig. 7F).

**Fig. 8:**
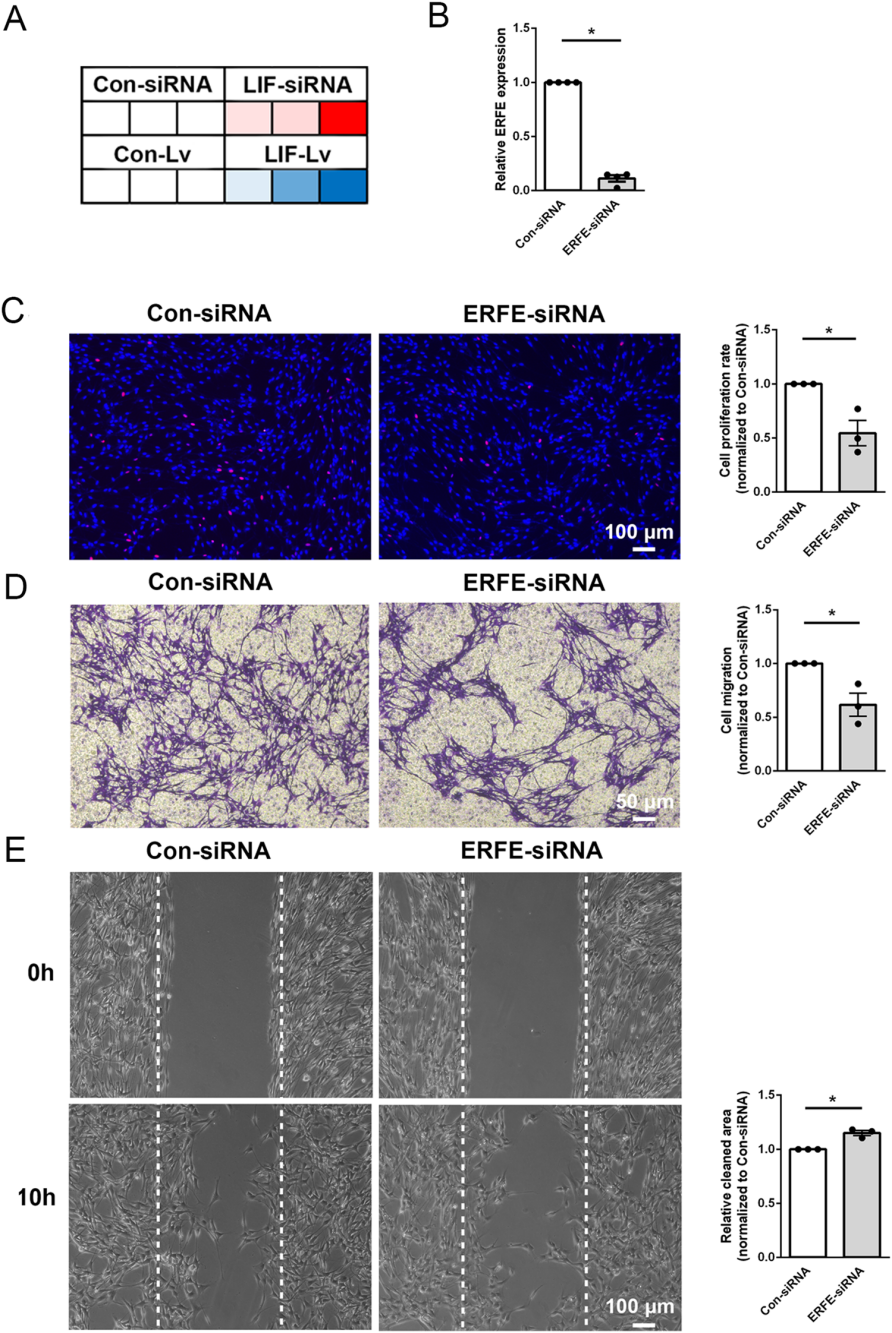
ERFE-siRNA suppresses Schwann cell proliferation and migration. **A** Heatmap of the relative expression levels of LIF mRNA in Schwann cells transfected with LIF-siRNA or LIF-overexpressing lentivirus (LIF-Lv). Red color indicates upregulation and blue color indicates downregulation. **B** Transfection of Schwann cells with ERFE-siRNA reduces the relative abundance of ERFE mRNA. **C** ERFE-siRNA transfection decreases Schwann cell proliferation. Red color indicates EdU-positive cells and blue color indicates nucleus. Scale bar indicates 100 μm. **D** ERFE-siRNA transfection decreases Schwann cell migration. Violet color indicates migrated cells. Scale bar indicates 50 μm. **E** ERFE-siRNA increases cleaned areas between scratches. Scale bar indicates 100 μm. Data were presented as means ± SEM. *p value <0.05 versus control-siRNA (Con-siRNA).

Among these overlaid differentially expressed genes, ERFE encodes erythroferrone and participates in various metabolic process. It implies that LIF may regulate ERFE expression and modulate the metabolism of Schwann cells. RT-PCR validation achieved consistent outcomes as RNA sequencing, indicating the accuracy of sequencing (Fig. 8A). Similar as Schwann cells transfected with LIF-overexpressing lentivirus, Schwann cells transfected with siRNA against ERFE (Fig. 8B) showed reduced proliferation rate (Fig. 8C) and inhibited migration ability (Fig. 8D, E). These findings indicate that LIF may affect the proliferation and migration of Schwann cells via negatively regulating ERFE.

## Discussion

Dysregulated genes after peripheral nerve injury may modulate various types of cells and contribute to nerve repair and regeneration. Consistent with the observations of the temporal expressions of LIF in the distal nerve segments after mice sciatic nerve crush or transection injury^18^, our work demonstrated that LIF is upregulated after rat sciatic nerve crush injury and is expressed in Schwann cells.

LIF, as a pleiotropic factor, execute opposite biological functions in diverse cell types^19^. For instance, when LIF was first cloned, LIF have been recognized as a differentiation-promoting factor in myeloid leukemic cells^20^, but a differentiation-suppressing factor in embryonic stem cells^21^. LIF was reported to be able to promote the survival, proliferation, and myelination of oligodendrocytes, the myelin-forming glial cells in the central nervous system^22–25^. Delivery of LIF to oligodendrocyte precursor cells promotes the differentiation of oligodendrocytes and increases the number of myelinated axons and the thickness of myelin sheaths^26^. The investigation of LIF on Schwann cells, the myelin-forming glial cells in the peripheral nervous system, was relatively less comprehensive. Our present study showed that different from its effects on oligodendrocytes, elevated LIF suppresses the proliferation and migration of primary cultured Schwann cells. The paradoxical roles of LIF on oligodendrocytes and Schwann cells may contribute to different regeneration capacity of injured central nerves and peripheral nerves. Notably, a previous study examined the effects of LIF on sNF96.2, Schwann cells collected from malignant peripheral nerve sheath tumor, and found that LIF inhibits the proliferation but enhances the migratory capacity of sNF96.2^27^. Therefore, LIF may exhibit distinctive effects not only on different cell types, but on different cellular status (e.g., tumor and normal cells).

Besides the investigation of the biological functions of LIF on cultured Schwann cells, we examined the effect of LIF on peripheral nerve regeneration using sciatic nerve injury models. Consistent with in vitro outcomes, accelerated Schwann cell proliferation and migration were observed after the direct application of LIF-siRNA to injured peripheral nerves. Proliferated and migrated Schwann cells towards the injure site contribute to boosted myelin and lipid debris removal and benefit the generation of a permissive microenviroment for regeneration. Detected accelerated axon elongation and myelin sheath formation, enhanced nerve conduction capacity, as well as improved recovery of motor function and sensory function reveal the essential role of LIF on peripheral nerve regeneration.

LIF generally affect cellular phenotype via stimulating ERK or STAT3 signaling pathways^28–30^. Here, we measured the abundances of p-ERK, ERK, p-STAT3, and STAT3 in Schwann cells transfected with LIF-siRNA or LIF-overexpressing lentivirus. The ratios of p-ERK to total ERK and p-STAT3 to total STAT3 were not significantly altered in LIF-overexpressing Schwann cells or decreased in LIF-knockdown Schwann cells, indicating that LIF may not influence Schwann cells through these canonical signaling pathways (Fig. S2). Sequencing data and functional evaluation revealed ERFE as a downstream effector of LIF. ERFE is a hepcidin suppressing hormone synthesized by erythropoietin (EPO)^31,32^. It has been demonstrated that EPO can promote the proliferation and myelin formation of Schwann cells and enhance the recovery of injured peripheral nerves^33–35^. Our present study demonstrates the benefiting roles of ERFE on Schwann cell proliferation and migration and thus indicates that EPO may encourage peripheral nerve regeneration via producing ERFE. An investigation of the association of LIF and EPO showed that in oligodendrocytes, LIF can inhibit EPO-induced, lipid transport and metabolism-associated genes^36^. Therefore, in Schwann cells, LIF may also negatively regulate EPO-induced ERFE and mediate peripheral nerve regeneration.

In summary, our findings demonstrate the role of LIF in Schwann cell behavior and peripheral nerve regeneration, elucidate the downstream effector of LIF, and may shed new light on the understanding of Schwann cell-associated molecular events during peripheral nerve repair and regeneration.

## Materials and Methods

### Rat sciatic nerve crush injury

Adult male Sprague-Dawley (SD) rats weighting 180–220 g were obtained from the Experimental Animal Center of Nantong University and subjected to sciatic nerve crush injury as previously described^37^. Sciatic nerve segments were collected from uninjured rats (designated as 0 h after nerve injury) and injured rats at 1 day, 4 days, 7 days, and 14 days after nerve injury. All procedures involving rats were approved ethically by the Administration Committee of Experimental Animals, Jiangsu, China and performed according to Institutional Animal Care Guidelines of Nantong University.

### Immunofluorescence staining

Sciatic nerve segments were fixed with 4% paraformaldehyde and cut with a cryostat (Leica Microsystems, Bensheim, Germany) into 10–12 µm thick sections. Tissue sections were exposed to primary antibodies anti-LIF (1:200, ab113262, Abcam, Cambridge, MA, USA) and anti-S100β (1:400, S2532, Sigma, St. Louis, MO, USA) followed by reaction with secondary antibodies Alexa Fluor 488 goat anti-mouse IgG (1:1000, Invitrogen, Carlsbad, CA, USA) and Cy3 sheep anti-rabbit IgG (1:1000, Invitrogen). Cell nucleus staining was conducted with DAPI (0100-20, SouthernBiotech, Birmingham, AL, USA). Tissue sections were visualized under ZEISS Imager M2 (Carl Zeiss Microscopy GmbH, Jena, Germany).

### Primary Schwann cell culture and transfection

Primary Schwann cells were collected from sciatic nerves of neonatal SD rats, purified with anti-Thy1.1 (1:1000, M7898, Sigma) and rabbit complement (Invitrogen, Carlsbad, CA, USA), and cultured in Dulbecco’s modified Eagle’s medium (DMEM; 10-013-CVR, Corning, NY, USA) containing 10% fetal bovine serum (FBS; 10099141c, Gibco, Grand Island, NY, USA), 1% penicillin and streptomycin (c0222, Beyotime, Shanghai, China), 2 μM forskolin (Sigma), and 10 ng/ml β-heregulin (HRG; R&D Systems Inc., Minneapolis, MN, USA) in a humidified 5% CO_2_ incubator at 37 °C. For LIF or EREF knockdown, cultured primary Schwann cells were transfected with siRNA segments targeting LIF (siRNA-1 sequence: GCTCATTCTGCACTGGAAA and siRNA-2 sequence: ATGCCAATGGGACAGAGAA), a siRNA segment targeting ERFE (sequence: TGAAGGAGTTCCAGTTGTT), or a non-targeting negative control (random sequence, RiboBio, Guangzhou, Guangdong, China) for 48 h using Lipofectamine RNAiMAX transfection reagent (Invitrogen). For LIF overexpression, cultured primary Schwann cells were transfected with LIF-overexpressing-lentivirus (pLenti-CMV-EGFP-P2A-LIF-3FLAG) or a negative control lentivirus (pLenti-CMV-EGFP-P2A-MCS-3FLAG) (OBiO, Shanghai, China) for 72 h.

### Real-time RT-PCR

Total RNAs were isolated from cultured Schwann cells and used as template for RNA quantification. RT-PCR was performed using SYBR Green Premix Ex Taq (TaKaRa) on a StepOne Real-time PCR System (Applied Biosystems, Foster City, CA, USA). The sequences of primer pairs were: LIF (forward) 5′- CGCCCAACATGACGGATTTC-3′ and (reverse) 5′- TTGTTGCACAGACGGCAAAG-3′; ERFE (forward) 5′-TGCATGAGCTCGGAGTCTAC-3′ and (reverse) 5′-TGAGTGCCACGTGAAGAGTG-3′; GAPDH (forward) 5′- CCTTCATTGACCTCAACTACATG-3′ and (reverse) 5′- CTTCTCCATGGTGGTGAAGAC-3′. Relative abundances of target genes were determined using the 2^-ΔΔCt^ method.

### EdU proliferation assay

Schwann cells were seeded on poly-l-lysine-coated 96-well plates at a density of 2 × 10^5^ cells/ml and exposed to 50 μM EdU for 24 h. A Cell-Light EdU DNA Cell Proliferation Kit (Ribobio) was applied to observe cellular proliferative status. Schwann cells were visualized under Leica Model DMi8 (Leica Microsystems CMS GmbH). Cell numbers were determined using Image-Pro Plus (Media Cybernetics, Rockville, MD, USA) and cell proliferation rate was calculated by dividing the number of EdU-positive cells to the number of total cells.

### Transwell migration assay

Schwann cells were suspended in DMEM and seeded on the upper chamber of a 6.5 mm Transwell with 8 μm pores (Costar, Cambridge, MA, USA) at a density of 4 × 10^5^ cells/ml. Schwann cells on the upper chamber were allowed to migrate towards the bottom chamber filled with 500 μl culture medium containing FBS. The upper chamber was taken out 24 h later and the upper surface of the upper chamber was cleaned with a cotton swab. The bottom surface of the upper chamber was stained with 0.1% crystal violet to visualize migrated cells under Leica DMI3000 B (Leica Microsystems). Migrated cells were quantified by crystal violet-stained areas or dissolving crystal violet with 33% acetic acid and measuring the intensity of the absorbance of crystal violet staining using a Synergy^TM^ 2 Multi-Mode Microplate Reader (BioTek, Burlington, VT, USA).

### Wound healing assay

Schwann cells were seeded on a mold chamber with a 1 mm wide insert placed on the bottom at a density of 2 × 10^5^ cells/ml. The 1 mm wide insert was removed after cell grew confluent. At 10–15 h after insert removal, cleaned areas were visualized under Leica DMI3000 B (Leica Microsystems). Cleaned areas were measured using Image-Pro Plus (Media Cybernetics) and relative cleaned areas were determined by dividing remaining cleaned area in the treated group to the remaining cleaned area in the control group.

### In vivo assay

Adult SD rats were subjected to sciatic nerve crush injury after anaesthetization. Rats were injected with 5 µl LIF-siRNA (siRNA-1) or a negative control (RiboBio) and 5 µl Matrigel (Corning) at the injury site immediately after nerve crush injury. EdU was intraperitoneal injected 24 h prior to nerve injury.

At 1 day, 4 days, and 7 days after nerve crush injury, sciatic nerve segments were collected, cut into 10–12 µm thick sections, and stained with EdU (C10340, Invitrogen), anti-S100β (1:400, S2532, Sigma), or anti-SCG10 (1:400, NBP1-49461, Novus Biologicals, Littleton, CO, USA) to observe Schwann cell migration and proliferation as well as axon elongation. Image J (National Institutes of Health, Bethesda, MA, USA) was applied to calculate relative fluorescence intensities.

At 2 weeks after nerve crush injury, rats were perfused with a fixative containing 1% paraformaldehyde and 1.25% glutaraldehyde. Sciatic nerve segments were fixed, embedded in Compound Tissue-Tek O.C.T Compound (Sakura FineTek, Torrance, CA, USA), and cut into ultrathin sections for transmission electron microscopy observations (JEOL Ltd., Tokyo, Japan).

At 1 week, 2 weeks, and 3 weeks after nerve crush injury, the recovery of motor function was assessed using the CatWalk XT system (Noldus Information Technology, Wageningen, the Netherlands) as previously described^38^. Paws were captured and 3D footprint intensities were measured. CatWalk mean intensity was calculated by dividing the absolute value of the difference of right hindpaw intensity and left hindpaw intensity to right hindpaw intensity to exclude inference factors such as body weight and paw size. SFI was calculated using the formula −38.3[(EPL−NPL)/NPL] + 109.5[(ETS−NTS)/NTS] + 13.3[(EIT−NIT)/NIT]−8.8 where EPL indicated the experimental paw length, NPL indicated the normal paw length, ETS indicated the experimental toe spread, NTS indicated the normal toe spread, EIT indicated the experimental intermediary toe spread, and NIT indicated the normal intermediary toe spread. A SFI value of −100 indicated nerve dysfunction while a SFI value of 0 indicated normal nerve function.

At 3 weeks and 4 weeks after nerve crush injury, CMAP responses were measured as previously described^39^. Signals were recorded by inserting electrodes into the mid-belly of gastrocnemius and electrical stimuli to the proximal and distal nerve stumps using Keypoint 2 portable electromyograph (Dantec, Denmark).

At 4 weeks after nerve crush injury, the recovery of sensory function was assessed by measuring mechanical pain thresholds as previously described^40^. 50% paw withdrawal latencies to mechanical stimulus were measured by rat hindpaw responses to Von Frey hairs (0.08–300 g; DanMic Global, LLC, San Jose, CA, USA).

### RNA sequencing and bioinformatic analysis

Schwann cell transfected with LIF-siRNA (siRNA-1), siRNA control, LIF-overexpressing lentivirus, or lentivirus control were subjected to RNA isolation and sequencing analysis. The cDNA libraries were sequenced on the Illumina sequencing platform by Genedenovo Biotechnology Co., Ltd (Guangzhou, Guangdong, China). Gene expression levels were determined using Fragments Per Kilobase of transcript per Million mapped reads (FPKM) method. Differentially expressed genes with a fold change >2 or < −2 and a false discovery rate (FDR) <0.05 as compared with corresponding controls were determined and enriched to KEGG pathways using OmicShare bioinformatic tools (Genedenovo). Overlapped differentially expressed genes were displayed in a Venn diagram using the Venny 2.1.0 online software^41^. Sequencing data were stored in SRA database with accession number SUB7176959 (PRJNA614579).

### Western blot

Protein samples isolated from cultured cells were loaded onto SDS-PAGE and transferred to PVDF membranes for protein quantification. Membranes were blocked with 5% BSA, incubated with primary antibodies p-STAT3 (phospho-Y705,1:1000, ZRB1000, Merck, Darmstadt, Germany), STAT3 (1:1000, ZRB1004, Merck), p-ERK (ERK1 (phospho-T202) + ERK2 (phospho-T185), 1:1000, ab201015, Abcam), ERK (1:10000, ab184699, Abcam), and β-actin (1:1000, Proteintech, Rosemont, IL, USA) followed by reaction with HRP-conjugated secondary antibodies (1:1000, Beyotime). Pierce^®^ ECL Western Blotting Substrate (ThermoFisher Scientific, Waltham, MA, USA) was applied for Western blot band detection. Images were visualized under Tanon Imaging Systems (Tanon, Shanghai, China).

### Statistic analysis

Summarized outcomes were demonstrated as means ± SEM from three to four experiments. Student’s *t*-test or ANOVA followed by Dunnett’s post hoc test was used for comparisons. A *p* value <0.05 was considered as significantly different.

## Supplementary information

Supplementary Figure Legends

Figure S1

Figure S2

## Data Availability

The datasets used and/or analyzed during the current study are available from the corresponding author on reasonable request.
