# Supplementary material for: Leukemia inhibitory factor regulates Schwann cell proliferation and migration and affects peripheral nerve regeneration
Source: Cell Death Dis. 2021 Apr 22;12(5):417. doi: 10.1038/s41419-021-03706-8 (PMC8062678; doi:10.1038/s41419-021-03706-8)
Supplement: Supplementary file 1 — Supplementary Figure Legends [file 41419_2021_3706_MOESM1_ESM.doc]

**Supplementary Figure Legends**

**Figure S1. Immunofluorescence staining of LIF in rat sciatic nerve segments.** The expressions and localizations of LIF in rat sciatic nerve segments at 0 hour, 1 day, 4 days, 7 days, and 14 days after nerve crush injury. Injured areas (proximal segments, crush sites, and distal segments) are labeled with dashed lines. Red color indicates LIF, green color indicates S100β, and blue color indicates nucleus. Boxed areas are demonstrated at a higher magnification on the right. Scale bars indicate 500 μm in main images and 100 μm in magnified images.

**Figure S2. Protein expressions of p-STAT3, STAT3, p-ERK, and ERK in LIF knockdown or over-expressed Schwann cells. (A)** Expressions of p-STAT3, STAT3, p-ERK, ERK, and β-actin in Schwann cells transfected with LIF-siRNA or siRNA control (Con-siRNA). **(B)** Expressions of p-STAT3, STAT3, p-ERK, ERK, and β-actin in Schwann cells transfected with LIF-overexpressing lentivirus (LIF-Lv) or lentivirus control (Con-Lv). **(C)** Quantification of the ratio of p-STAT3 and STAT3 in Schwann cells transfected with LIF-siRNA or siRNA control. **(D)** Quantification of the ratio of p-ERK and ERK in Schwann cells transfected with LIF-siRNA or siRNA control. **(E)** Quantification of the ratio of p-STAT3 and STAT3 in Schwann cells transfected with LIF-overexpressing lentivirus or lentivirus control. **(F)** Quantification of the ratio of p-ERK and ERK in Schwann cells transfected with LIF-overexpressing lentivirus or lentivirus control. Data are presented as means ± SEM.
