# Supplementary figures and images for: Leukemia inhibitory factor regulates Schwann cell proliferation and migration and affects peripheral nerve regeneration
Source: Cell Death Dis. 2021 Apr 22;12(5):417. doi: 10.1038/s41419-021-03706-8 (PMC8062678; doi:10.1038/s41419-021-03706-8)

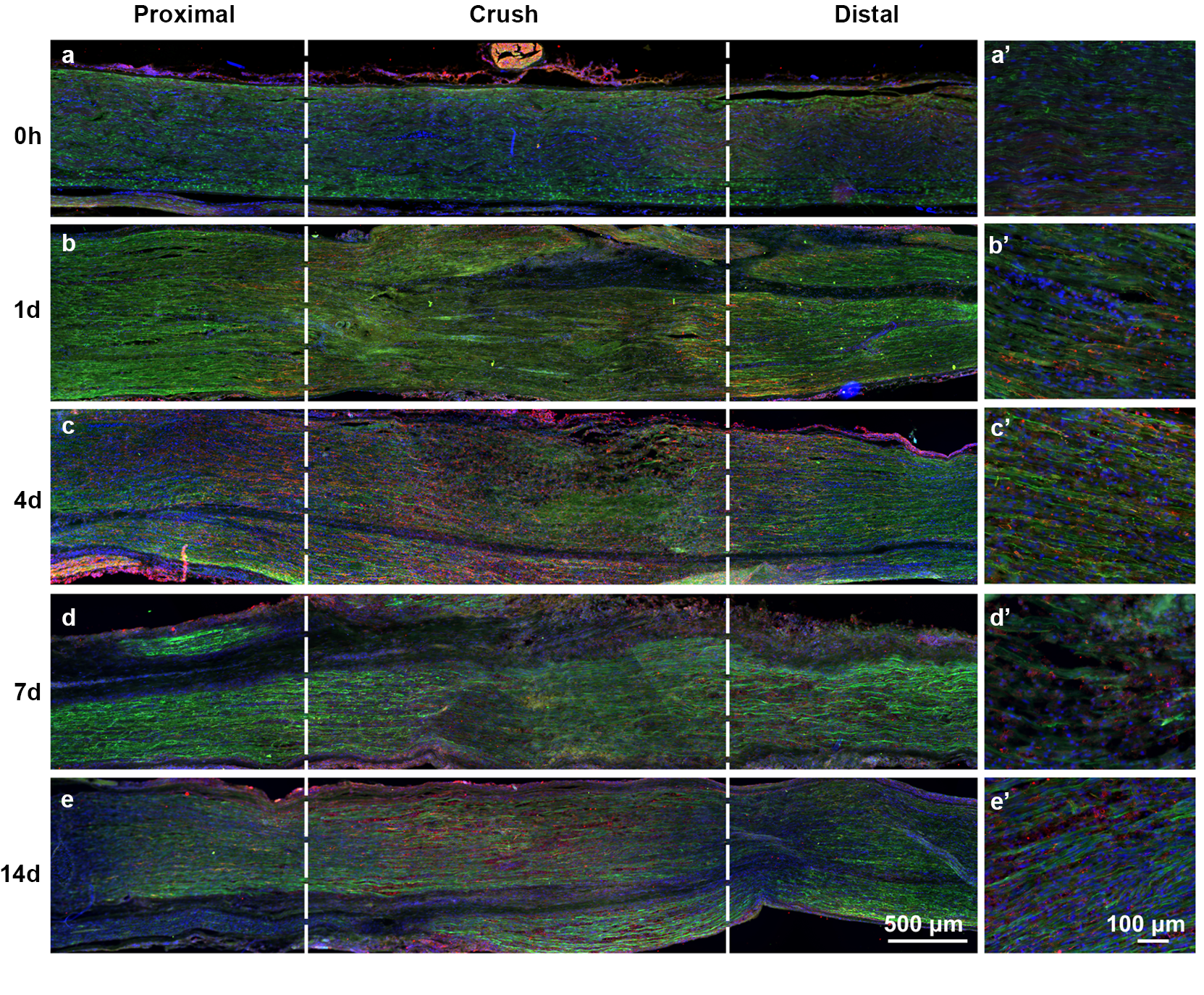

Supplement: Supplementary file 2 — Figure S1 [file 41419_2021_3706_MOESM2_ESM.tif]

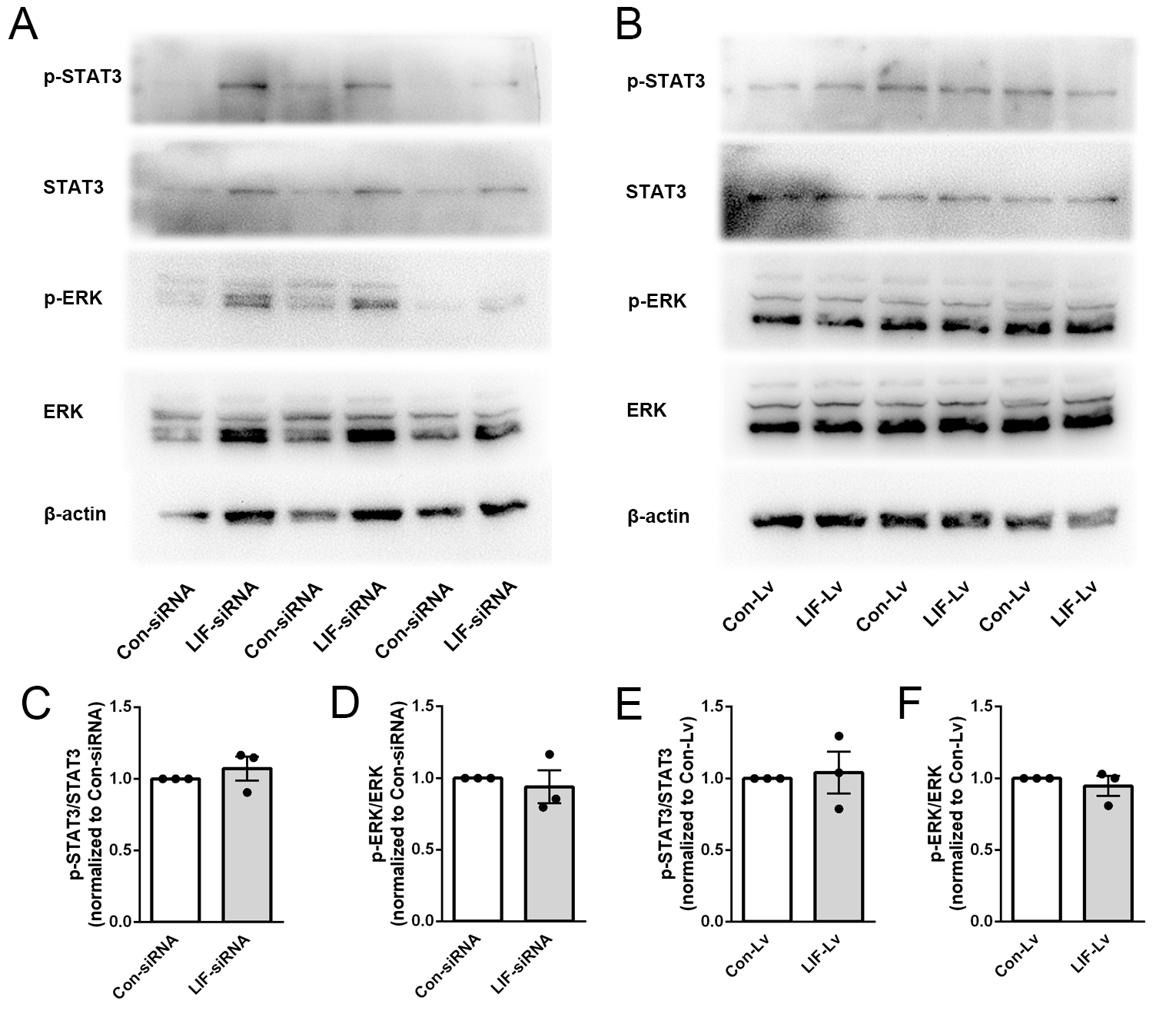

Supplement: Supplementary file 3 — Figure S2 [file 41419_2021_3706_MOESM3_ESM.tif]
